# Hybrid Gibbsite Nanoplatelet/Cellulose Nanocrystal Multilayered Coatings for Oxygen Barrier Improvement

**DOI:** 10.3389/fchem.2019.00507

**Published:** 2019-07-17

**Authors:** Maud Chemin, Laurent Heux, David Guérin, Laura Crowther-Alwyn, Bruno Jean

**Affiliations:** ^1^Univ. Grenoble Alpes, CNRS, CERMAV, Grenoble, France; ^2^Centre Technique du Papier, Functional Products and Surfaces, Grenoble, France

**Keywords:** oxygen barrier, layer-by-layer (LbL), thin films, gibbsite nanoplatelets, cellulose nanocrystals

## Abstract

We have investigated the ability of multilayered hybrid thin films of cellulose nanocrystals (CNCs) and gibbsite nanoplatelets (GNPs) to be built by the layer-by-layer (LbL) technique onto substrates selected for packaging applications, and to improve the oxygen barrier properties. Using complementary structural characterization techniques, namely atomic force microscopy, ellipsometry, and spectral reflectance, we show that when deposited onto model silicon substrates these hybrid films were homogenous and of reduced porosity, and were comprised of alternately deposited monolayers of GNPs and CNCs. The successful deposition of such homogeneous and dense hybrid thin films onto various types of flexible substrates showing different chemical compositions, hydrophilicity, and surface morphology, ranging from cardboard to smart paper, polyethylene (PE) films, and PE-coated cardboard was also confirmed by scanning electron microscopy observations. In view of the diversity of these substrates we could confirm the remarkable robustness of such a deposition process, likely due to (i) the adaptability of the LbL assembling technique and (ii) the strong electrostatic and hydrogen bonding interactions between GNPs and CNCs. The measurement of the oxygen transmission rate (OTR) at 23°C and 50% RH showed that the oxygen barrier properties of the bare substrates could be significantly improved (e.g., 75% decrease of the OTR) after the deposition of such thin (<100 nm) multilayered hybrid films. This lowered permeability was tentatively attributed to the highly tortuous morphology of the coating, acting to impede the gas diffusion. These partially biosourced very thin films stand as good candidates for using as coatings showing high oxygen barrier performance.

## Introduction

Food packaging is a constantly growing market, which is however still mainly based on the use of non-renewable and non-biodegradable petroleum-derived species. To overcome this situation, which causes considerable environmental issues, a world-wide research effort is undertaken to develop eco-friendly packaging materials containing building blocks of renewable origin. In this context, as summarized in numerous recent reviews, nanocelluloses have emerged as a promising class of bio-based colloids, able to fulfill the requirements for the production of thin films and coatings, adapted to packaging applications, in particular exhibiting high oxygen barrier, optical transparency and mechanical resistance (Azeredo et al., 2017; Ferrer et al., 2017; Hubbe et al., 2017; Thomas et al., 2018; Qin et al., 2019). Nanocelluloses comprise both (i) the slender flexible nanofibrils (CNFs) extracted following a mechanical disintegration of cellulose fibers coupled with enzymatic and/or chemical treatments and (ii) the shorter rigid cellulose nanocrystals (CNCs) usually derived from partial sulfuric acid hydrolysis of any cellulose source (Klemm et al., 2011, 2018; Moon et al., 2011). Both CNFs and CNCs, which are endowed with a rather low density and strong mechanical properties, are now commercialized in large quantities. While the flexible nature of the CNFs makes them highly appropriate for film formation, a high degree of nanofibrillation is needed if one wants to obtain a low porosity and low permeability of these so-called nanopapers. However, such requirements are not always met by industrial products, which may contain high fractions of microfibers. To the opposite, CNCs often yield brittle films when casted, but thanks to their tunable cellulose-source-dependent aspect ratio and true homogeneous nanometric size (typically a few hundreds of nm in length and 3–30 nm in cross-section), they can be used as major ingredients to prepare thin functional surface coatings deposited onto cellulosic or non-cellulosic substrates (Li et al., 2013).

As far as the deposition method is concerned, the layer-by-layer (LbL) assembly technique first introduced by Iler and then developed by the Decher group stands out as a highly convenient technique to build nanostructured multilayered thin films associating two species, providing that they exhibit mutual attractive interactions (Iler, 1966; Decher et al., 1992; Decher and Schlenoff, 2012). Initially introduced by Iler with colloids, the method then focused on polyelectrolytes with opposite charges, and has now been expanded to a wide variety of building blocks. In particular, it was shown that CNCs and bio- or synthetic polymers could be assembled using the LbL technique in multilayered thin films with a very high degree of structural control, which cannot be reached with other deposition techniques (Martin and Jean, 2014). In a recent work, we have replaced the polymer component by positively charged inorganic gibbsite nanoplatelets (GNPs) to form innovative hybrid all-nanoparticles (GNPs/CNCs) thin films (Martin et al., 2017). The choice of GNPs, which show structural similarities with clay nanoparticles (e.g., montmorillonite or vermiculite), was based on their property to impede the diffusion and permeation of gas molecules, as shown in numerous studies (Priolo et al., 2010, 2012, 2015; Wu et al., 2012; Rhim et al., 2013; Song et al., 2016; Qin et al., 2019). In the work by Martin et al., a structural investigation based on the use of atomic force microscopy (AFM) and neutron reflectivity showed that the growth and density of GNPs/CNCs films could be tuned over a wide range during their preparation by varying the ionic strength of the CNCs suspension and the film drying protocol (Martin et al., 2017). Specifically, by building the films under aqueous conditions with no added salt in the suspensions and drying the films at the end of the process yielded very thick porous slabs. On the other hand, drying the film after each layer deposition and adding 10 mM of monovalent salt in the CNCs suspension led to the production of thin, dense and well-stratified multilayered films.

The present study addresses the possibility to use our established protocol for such multilayered architecture as an efficient coating to improve the oxygen barrier properties of different cellulosic and non-cellulosic substrates possessing different chemical compositions, hydrophilicity, mechanical properties, and surface morphology. We first describe the building of GNPs/CNCs films onto model silicon substrates (inherently covered by a thin silicon oxide layer) using the LbL assembly technique, characterizing the films by AFM, ellipsometry and light reflectance. Then, the ability of such hybrid thin films to be deposited on various substrates was investigated and the resulting structures were characterized by scanning electron microscopy. The effect of the deposition of GNPs/CNCs multilayered films on the oxygen transmission rate was finally investigated to probe the ability of these thin partially biosourced coatings for improving oxygen barrier properties.

## Experimental Section

### Materials

#### Chemicals

Cotton linters were provided by Buckeye Cellulose Corporation and used as the cellulose source for CNCs without any further purification. Polyethyleneimine (PEI, *Mw* ≈ 25,000), poly(allylamine hydrochloride) (PAH, *Mw* ≈ 50,000), and poly(sodium 4-styrene sulfonate) (PSS, *Mw* ≈ 70,000) were purchased from Sigma-Aldrich and used at 2, 4, and 4 g/L, respectively. Aluminum isopropoxide (>98%, *M* = 204.25 g/mol) and aluminum sec-butoxide (>98%, *M* = 246.33 g/mol) were purchased from TCI and used without any further purification. NaCl, HCl 37% and H_2_SO_4_ 96% were purchased from Prolabo, Fisher Scientific, and Acros Organics, respectively. Ethanol and chloroform were purchased from Biosolve. Demineralized water was systematically used.

#### Substrates

Four different flexible substrates of interest were used for film deposition: an uncoated kraft cardboard of 225 g/m^2^ produced by StoraEnso (CKB Nude™), a polyethylene-coated cardboard produced by StoraEnso (Cupforma Natura 2PE) and composed of a base board of 232 g/m^2^ coated on both sides with polyethylene (PE, 12 and 15 g/m^2^ on topside and reverse side, respectively), a low density virgin PE substrate of 92.5 g/m^2^ from RAJA (sheath 30B) and a smart paper of 190 g/m^2^ from Felix Schoeller (pe:smart paper type 1). This last substrate was composed of a raw paper coated on both sides with resin and with a hydrophilic primer layer on topside. It was developed in the framework of the European project Autonomous Printed Paper Products for functional Labels and Electronics (A3Ple). Additionally, one-side polished <001> silicon wafers (Sil'tronix ST) were used as model solid substrates.

#### Cellulose Nanocrystals (CNCs)

Cellulose nanocrystals were prepared from the sulfuric acid hydrolysis of cotton linters as initially reported by Revol et al. (1992). Briefly, the cotton linters were treated with 64 wt.% sulfuric acid for 30 min at 60°C, cooled by ice addition and washed by four centrifugation/redispersion cycles. The resulting suspension was dialyzed against deionized water until the conductivity of the dialysis bath reached the conductivity of deionized water and sonicated twice for 3 min with a Branson model 450 sonifier at 30% amplitude. After these treatments, the suspension was filtered through 8 μm and then 1 μm cellulose nitrate membranes (Sartorius). The resulting 2.3 wt.% CNC suspension was concentrated by ultrafiltration using 10,000 kDa ultrafiltration membranes from Millipore to 3.9 wt.% and 10 mM of NaCl was added to the CNCs suspension, which was used for multilayered film build up. To avoid any microbial spoilage, about 1 mL chloroform per liter was added to the suspensions, which were stored at 4°C.

#### Gibbsite Nanoplatelets (GNPs)

Gibbsite nanoplatelets were obtained by a hydrothermal treatment of 0.08 M aluminum isopropoxide and 0.08 M aluminum sec-butoxide in 0.09 M hydrochloric acid (Wijnhoven et al., 2005). After a 10 day dissolution step, the suspension was heated for 72 h at 84°C in an oven (to limit the growth of boehmite nanorods at higher temperature) and then dialyzed against deionized water. The resulting 0.8 wt.% suspension was concentrated by ultrafiltration using 10,000 kDa ultrafiltration membranes (Millipore) to 4.8 wt.%. The pH of the GNPs suspension was set at 5.9 using hydrochloric acid (0.1 M). To avoid any bacterial contamination, some chloroform was added to the suspension, which was stored at 4°C.

### Layer-by-Layer Assembly

The Si wafers solid substrates were cleaned with ethanol, followed by rinsing with water. Prior to hybrid film deposition using the LbL assembly process, all substrates, i.e., solid model or flexible, were subjected to a plasma cleaning treatment (PELCO easiGlow™, 0.39 mbar, 15 mA, 25 s) to get rid of organic contaminants and possibly confer negative charges to the surface. Before the deposition of the first GNPs layer, a multilayered “primer” composed of a PEI/PSS/PAH/PSS polyelectrolyte stack was laid on the substrate by sequential dipping into the corresponding solution for 20 min for PEI and 15 min for PSS and PAH, with intermediate rinsing steps of 5 min in water. Hybrid thin films were then assembled using a dipping LbL process as illustrated in **Figure 2**. The negatively charged substrate was first immersed in the positively-charged GNPs suspension for 5 min, then the substrate was rinsed for 5 min with distilled water and the substrate was immersed in the negatively charged CNCs suspension for an identical time and finally rinsed for 5 min. The rinsing steps are required to remove loosely bound nanoparticles and ensure that only strongly interacting particles are adsorbed. This cycle was repeated to reach the desired number of deposited bilayers, *n*, where a bilayer is defined as a layer of GNPs plus a layer of CNCs. Samples were dried using gentle air blowing after each dipping/rinsing step, i.e., after each layer deposition.

For solid silicon model substrates, samples with three different numbers of bilayers, *n* = 4, 7, and 11, have been prepared. For flexible substrates that were used to evaluate the potential of the film as an oxygen barrier coating, samples with *n* = 1, 4, 7, and 7.5 were prepared. *n* = 7.5 corresponds to the samples with *n* = 7 plus an additional layer of GNPs only. For these samples, each experiment was realized in triplicate to allow three oxygen permeability measurements.

The notation (GNPs/CNCs)_n_ designates a multilayered film containing *n* number of deposited (GNPs/CNCs) bilayers. The polyelectrolyte primer layer was omitted in the notation for clarity.

### Methods

#### Zeta Potential

The zeta potential of CNCs and GNPs was measured from 0.1 wt.% suspensions by electrophoresis coupled with laser Doppler velocimetry using a Malvern NanoZS instrument. Samples were measured in 10 mM NaCl. Data were averaged over three measurements, each of them comprising 10 sub-runs.

#### Transmission Electron Microscopy

Drops of *ca*. 0.001 wt.% GNPs or CNCs suspensions were deposited onto glow-discharged carbon-coated TEM grids. After 2 min, the liquid in excess was wicked away with a filter paper and, for CNCs only, prior to drying, a drop of 2% uranyl acetate was deposited on the specimen. After 2 min, the stain in excess was blotted off and the remaining thin liquid film was allowed to dry. The specimens were observed using a Philips CM200 electron microscope operated at 80 kV. The images were recorded with a TVIPS F216 TemCam camera (2,040 × 2,040 pixels).

#### Atomic Force Microscopy

AFM height images were recorded at randomly selected surface positions in peak force mode using a Dimension Icon instrument (Bruker, Santa Barbara, CA). The cantilevers Scanassist-Air (Bruker, Santa Barbara, CA) used were triangular and had a force contact of 0.4 N/m and a resonance frequency of 70 kHz at tip scan rates of 1 Hz. AFM images were processed using the flattening function of the Gwyddion software, and the RMS roughness was calculated on 5 × 5 μm^2^ images.

#### Scanning Electron Microscopy

Prior to observations, samples were coated with 2–3 nm Au/Pd using a Baltec MED 020 apparatus. Secondary electron images of the specimens were recorded with a FEI Quanta 250 scanning electron microscope (SEM) equipped with a field emission gun and operated at 2 kV.

To obtain images of cross-sections, samples were prepared as follows. A piece of the sample (about 0.3 by 0.6 cm^2^) was placed between two pieces of polystyrene so as to sandwich the flexible sample and prevent deformation. This assembly was then placed in a sample holder, which was introduced in the cryo-ultramicrotome (Leica UC6 instrument) tank cooled at −110°C. The cooled samples were cut to obtain sharp cross-sections and brought back to room temperature by blowing with compressed air to avoid condensation of water on the surface.

#### Ellipsometry

Ellipsometric measurements were performed using an imaging ellipsometer EP3-SE (Nanofilm Technology GmbH, Germany). Experiments were performed *ex situ* under air conditions at a wavelength ranging from 379 to 809 nm (Xenon lamp was used as light source) at three different angles of incidence: 65, 70, and 75°. The instrument was used in total internal reflection mode and both the intensity and the phase changes of the reflected light were monitored and converted into two ellipsometric angles Ψ and Δ. The data were acquired and evaluated using the EP3View V235 Software (Nanofilm, Germany). Optical modeling was performed using the EP4Model 1.0.1 software (Nanofilm, Germany). All thicknesses were measured at only one spot of the sample with four nulling zones, leading to 0.1–0.5 nm and 0.006 accuracy for respectively the thickness and the refractive index.

The fit model used was composed of four layers: silicon substrate, a SiO_2_ layer of 1 nm, a primer layer, and a transparent (GNPs/CNCs)_n_ film as a Cauchy layer without extinction (*k* = 0) with a refractive index n_*film*_calculated as:

(1) $$\begin{array}{l} n_{film}\left(\lambda \right)=A+\frac{B}{\lambda^{2}} \end{array}$$

Least-square fitting of the experimental data with adequate optical models allowed us to determine the refractive index as well as the thickness of the film layer. Then, according to the effective medium approximation model (Xie et al., 2006), the particle volume fraction could be calculated as:

(2) $$\begin{array}{c} \Phi \left(\text{nanoparticles}\right)=\frac{\text{A}\left(\text{film}\right)-1}{\text{A}\left(\text{nanoparticles}\right)-1} \end{array}$$

Note that this equation is only valid for *k* = 0. Moreover, A_nanoparticles_ was chosen equal to 1.56 corresponding to GNPs and CNCs.

Before measurements on GNP/CNC coated samples, the thickness of the primer layer was measured in exactly the same condition with A(primer) = 1.435 and B(primer) = 0 corresponding to the average refractive index of each polymer composing the primer layer. The primer layer thickness was then calculated to be equal to 6.6 nm.

#### Spectral Reflectance

SR measurements were performed using a F20 Thin-Film Analyzer (Filmetrics, USA). Experiments were performed at a wavelength ranging from 190 to 1,100 nm. The data were acquired and evaluated by the FILMeasure software, using standard SiO_2_ layer on silicon substrate. The Filmetrics technique allowed determining the thin-film thicknesses by measuring the light that is reflected perpendicular to the film surface over a wide range of wavelengths. It then analyses these data by comparing it to a series of calculated reflectance spectra with an accuracy of 2 nm. All thicknesses given are an average value of 10 measurements performed at different spots on each sample. The thickness deviation was measured as 0.3, 2.4, 0.6, and 1.1 nm for *n* = 0, 4, 7, and 11, respectively.

#### Oxygen Transmission Rate Measurement

The OTR was measured according to the Centre Technique du Papier (CTP) internal method, adapted from ASTM F2714-08—Oxygen Headspace Analysis of Packages Using Fluorescent Decay. The measurements were performed with a fiber optic oxygen transmitter (Fibox 4 from PreSens Precision Sensing GmbH, Germany). The oxygen sensor in the optical window of the upper chamber was read out via a polymer optical fiber, which is connected to an oxygen transmitter. The upper chamber was flushed with oxygen free medium (nitrogen, 23°C, 0% relative humidity) while the lower chamber was flushed with a medium of known oxygen concentration, here conditioned air at 23°C and 50% relative humidity. The tested material was fixed between these chambers. The specific exchange area was fixed at 7.07 cm^2^ using an aluminum mask (tape 1456 from 3M). The oxygen transmission rate of the tested material was calculated from the increase in oxygen concentration over time in the upper chamber, in volume of oxygen per area, gas pressure and time (cm^3^/m^2^·d·bar). The experiments were carried out once on each coated substrate giving an averaged value over three measurements.

## Results and Discussion

### Nanoparticles Characterization

**Figure 1:**
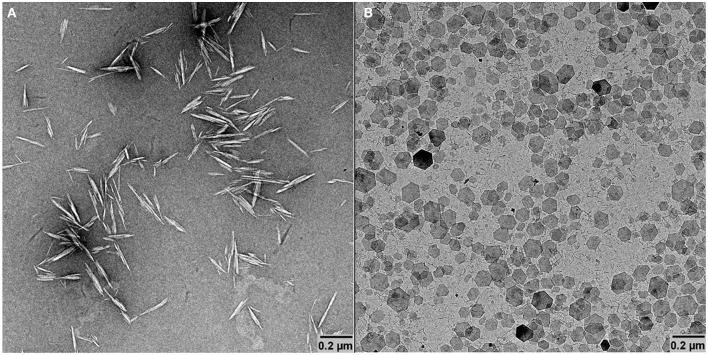
TEM micrographs of CNCs **(A)** and GNPs **(B)**.

The basic structural and charge properties of the two types of nanoparticles used to build the hybrid CNCs/GNPs thin films were first characterized. As shown in Figure 1A, the produced cotton CNCs are rod-like particles with a length between 100 and 300 nm and a width between 10 and 30 nm, each particle being a fascicle of a few parallel elementary subunits, in line with literature reports (Elazzouzi-Hafraoui et al., 2008). AFM and small angle neutron scattering results from the literature further show that the height of cotton CNCs is ~6 nm (Elazzouzi-Hafraoui et al., 2008; Cherhal et al., 2015; Martin et al., 2017). The TEM micrograph in Figure 1B shows that GNPs appear as hexagonal platelets of 105 nm average diameter. In our previous work, the height of these platelets, ~ 4 nm, was extracted from a statistical analysis of AFM topography images (Martin et al., 2017). Zeta potential measurements indicated that CNCs were negatively charged with a value of −40 ± 2 mV and that GNPs were positively charged with a value of +52 ± 3 mV. In addition, conductometric titration of CNCs suspensions gave a charge content of about 260 mmol kg^−1^, which corresponds to a sulfur content of 0.69% and a charge density of 0.5 e^−^/nm^2^, assuming all charges stem from sulfate groups. According to the literature, the surface charge density of GNPs is 5 +/nm^2^ (Wierenga et al., 1998). Note that the pH of the CNCs suspension was 2.2, ensuring CNCs to be negatively charged. Ester-sulfate groups resulting from the sulfuric hydrolysis are indeed in their acidic form under these conditions. For GNPs, the pH is a sensitive parameter: Below pH 4 the GNPs dissolve in water; between pH 4 and pH 7 the GNPs are positively charged but above pH 7, the particle edges become negatively charged and gelation may occur (Wierenga et al., 1998). Accordingly, to ensure that both types of particles exhibit opposite charges, the pH of the GNPs suspension was adjusted to 5.9.

### Building Hybrid Films on Model Substrate

**Figure 2:**
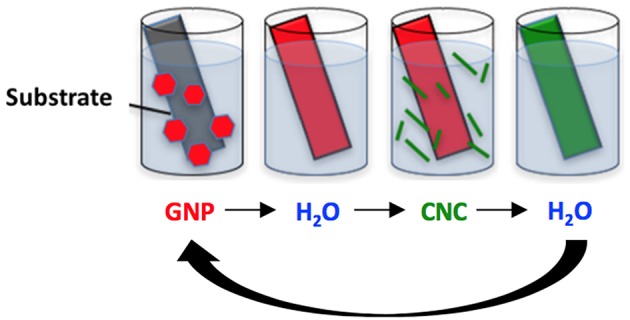
Schematic representation of the layer by layer assembly process.

**Figure 3:**
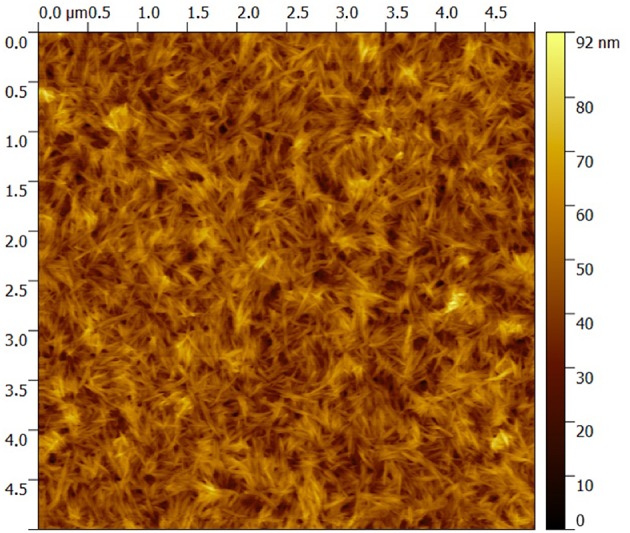
5 × 5 μm^2^ AFM topography image of a (GNPs/CNCs)_4_ multilayered film deposited on a Si wafer.

GNPs/CNCs multilayers were first assembled on silicon wafers as smooth and solid model substrate using the LbL assembly process, as illustrated in Figure 2. The structural properties of the resulting films were investigated by AFM, ellipsometry and spectral reflectance. AFM topography images performed on (GNPs/CNCs)_4_, (GNPs/CNCs)_7_, and (GNPs/CNCs)_11_ films show surfaces densely covered by CNCs (Figure 3). As shown in our previous work, this observation confirms that the used LbL process conditions led to the formation of homogeneous films. Strikingly, no underlying platelets can be distinguished, suggesting that the interactions between the two types of particles are particularly effective to allow a full coverage of a GNPs layer by a CNCs layer. In fact, the interaction between CNCs and GNPs is strong enough to allow for a short dipping time between the layer deposition: this time could be reduced to 5 min, as opposed to 15 min in our previous study. In Figure 3, the CNCs appear randomly oriented and their RMS rugosity was calculated equal to 8.2 ± 1.1 nm. As shown in Supplementary Figure 1, the GNPs-terminated films showed a very good coverage of the underlying CNCs layer by the subsequent GNPs deposition.

**Figure 4:**
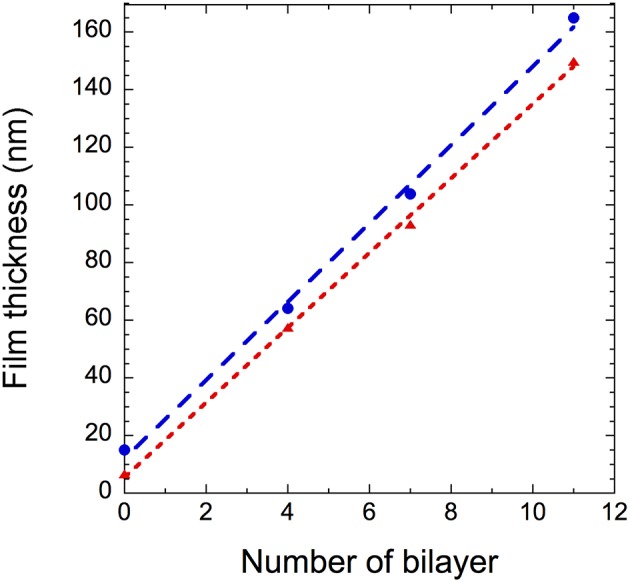
(GNPs/CNCs)_n_ film thickness as a function of the number of bilayers deposited on a Si wafer using spectral reflectance (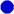) and ellipsometry (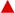). Dashed lines are linear fits to the data.

**Figure 5:**
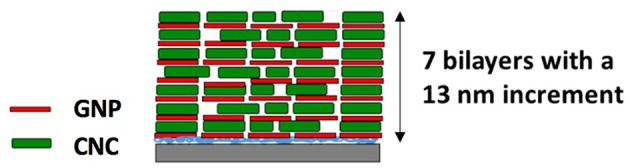
Schematic representation of the likely structure of a (GNPs/CNCs)_7_ hybrid film.

The thickness of the films determined by ellipsometry and spectral reflectance is plotted in Figure 4 as a function of the number of deposited bilayers. The thickness increases linearly with *n*, irrespective of the technique used, showing a successful film growth. The thickness increment per bilayer is equal to 13 nm. Knowing that the thicknesses of nanoparticles are around 4 and 7 nm for GNPs and CNCs, respectively, one can conclude that each deposition corresponds to a single layer of each nanoparticle type. Moreover, the measured thicknesses are in total agreement with our previous work, where the film thicknesses were measured using AFM and neutron reflectivity (Martin et al., 2017). In addition, ellipsometry measurements allowed us to calculate the particle volume fraction Φ in the films from their refractive index (Equation 2). Table 1 summarizes the values of refractive index and corresponding particle volume fraction for *n* = 4, 7, and 11. Φ is close to 0.85 for all *n* values, showing that the films are very dense. Note that in our previous work a Φ value of nearly 0.8 was deduced from neutron reflectivity (Martin et al., 2017). This comparison therefore allows us to validate the present ellipsometry data. The linear growth together with the constant particle volume fraction suggest that the hybrid films built on model Si wafers exhibit the same internal structure irrespective of the thickness: they consist of repeated dense bilayers, leading to smooth films of low porosity and likely stratified as schematized in Figure 5.

**Table 1:** Thickness, refractive index, and particle volume fraction of (GNP/CNC)_n_ determined by ellipsometry.

Number of bilayers	Thickness (nm)	Refractive index (A)	Refractive index (B)	*Φ_*nanoparticles*_*
4	57.6	1.481	6,917	0.83
7	93.2	1.512	6,712	0.90
11	149.9	1.492	9,932	0.86

### Growth and Structure of the Films on Various Substrates

The aforementioned results demonstrate our ability to build dense hybrid all-nanoparticle GNPs/CNCs multilayered films on Si wafer. In the next step, we have investigated the possibility to build these films on various substrates, i.e., kraft cardboard, PE-coated cardboard, PE-LD film, and smart paper, exhibiting different chemical compositions, hydrophilicity, mechanical properties, flexibility and surface roughness.

**Figure 6:**
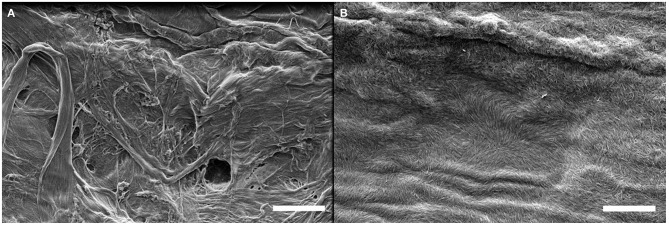
SEM images of the kraft cardboard substrate before **(A)** and after **(B)** deposition of a (GNPs/CNCs)_4_ multilayered film. Scale bar: 2 μm.

**Figure 7:**
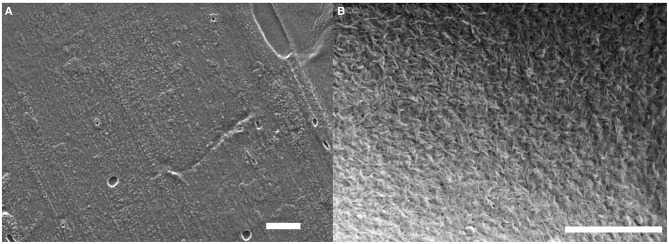
SEM images of the PE-coated cardboard substrate before **(A)** and after **(B)** deposition of a (GNPs/CNCs)_4_ multilayered film. Scale bar: 2 μm.

**Figure 8:**
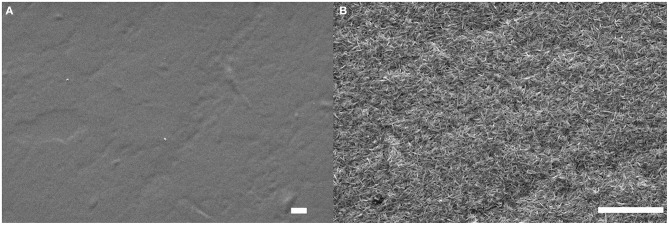
SEM images of the smart paper substrate before **(A)** and after **(B)** deposition of a (GNPs/CNCs)_4_ multilayered film. Scale bar: 2 μm.

The same LbL process was used on these four substrates and SEM imaging was performed to observe the surface of the deposited film. SEM micrographs of the substrates before and after the deposition of a (GNPs/CNCs)_4_ film for kraft cardboard, PE-coated cardboard, smart paper and PE-LD film are shown in Figures 6–8 and Supplementary Figure 2, respectively. A successful deposition is observed on all studied substrates. The observations are similar to those with the Si wafers, showing very dense and homogeneous films with a complete coverage of GNPs by CNCs. Since the (GNPs/CNCs)_n_ films were very thin (<200 nm), the surface topography of the raw substrate was preserved. Identical dense CNCs top layers were obtained for hydrophobic (PE-coated cardboard and PE-LD films) as well as for hydrophilic (kraft cardboard and smart paper) substrates, smooth (PE-coated cardboard, PE-LD films and smart paper), rough (kraft cardboard) surfaces and very flexible (PE-LD films and smart paper) together with more rigid (kraft cardboard and PE-coated cardboard) samples. These results confirm the exceptional robustness of the deposition and building process, which is due not only to the versatility of the LbL assembly technique but also to the strong interactions between CNCs and GNPs that even allow using relatively short dipping times.

In addition, as shown in Supplementary Figures 3, 4, GNPs-terminated coatings on PE-LD and kraft cardboard substrates exhibit a fairly regular and dense paving of the surface by the inorganic platelets, which is in line with the observations on model substrates. The inherent higher roughness of the commercial substrates therefore does not seem to impede the propensity of GNPs to densely cover the underlying CNCs and to lay flat on the surface.

**Figure 9:**
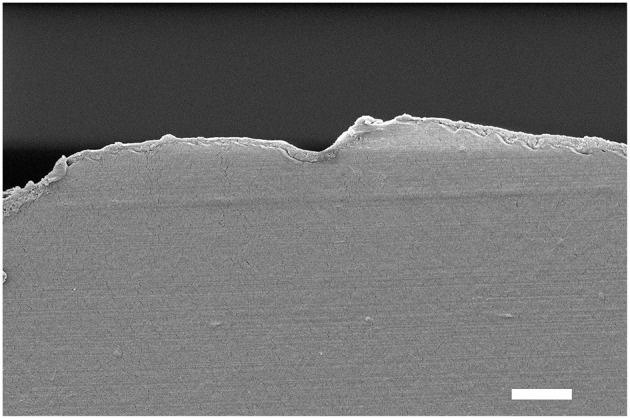
Cross-section SEM image of the smart paper substrate after deposition of a (GNPs/CNCs)_7_ multilayered film. Scale bar: 2 μm.

The attempts to obtain sharp cross-sections of the films proved difficult, possibly due to the mismatch in composition and mechanical properties between the substrate and coating. Nevertheless, exploitable images could be recorded for (GNPs/CNCs)_4_ films onto kraft cardboard and PE-LD and (GNPs/CNCs)_7_ films onto PE-coated cardboard and smart paper substrates (Supplementary Figures 5–7 and Figure 9, respectively). These images first showed void-free contact between the coating and the external surface of the different substrates, irrespective of their roughness or hydrophilic or hydrophobic character. In addition, it could be observed in each case that in the direction perpendicular to the film surface, homogeneous and dense (GNPs/CNCs) coatings were deposited. The thickness of the coating is constant along the cross-section and follows in a smooth and continuous manner the morphology of the underlying substrate. Quantitatively, as shown in Supplementary Figure 8, the thickness values that were measured are in close agreement with the values obtained on model Si surfaces, suggesting a similar film architecture (i.e., a coating composed of superimposed nanoparticle-monolayers) and indicating a linear thickness increase of the coating on the commercial substrate with the number of deposited layers.

### Oxygen Barrier Properties

**Figure 10:**
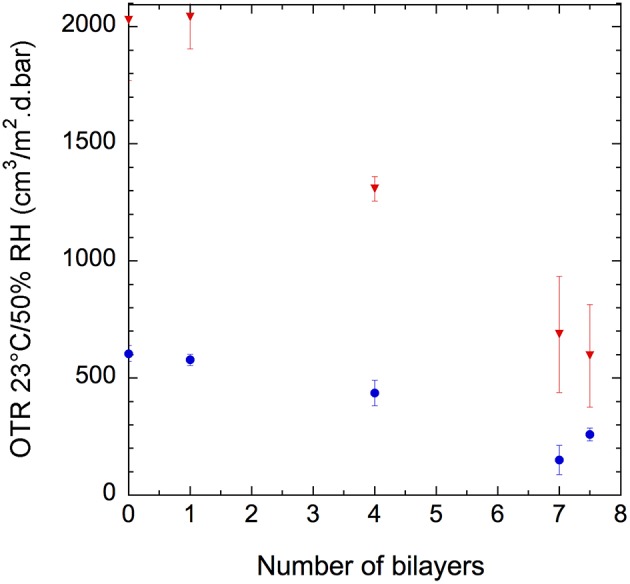
Oxygen transmission rate of (GNPs/CNCs)_n_-coated PE-coated cardboard (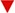) and smart paper (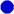) substrates as a function of the number of deposited bilayers, *n*, at 23°C and 50% RH.

In Figure 10, we present the effect on the oxygen barrier properties of the deposition of (GNPs/CNCs)_n_ thin films onto PE-coated cardboard and smart paper substrates as a function of bilayers number. It must be noted that measurements were performed with a fiber optic oxygen transmitter (at 23°C using conditioned air at 21% oxygen and 50%RH as test gas, and dry nitrogen as carrier gas. Results indicate that for both substrates, the deposition of one bilayer does not show any positive or negative effect on oxygen transmission. Nevertheless, with four bilayers a positive effect started to occur. Indeed, for smart paper and PE-coated cardboard, the OTR value, respectively dropped down by 20% and 36%, after the deposition of (GNPs/CNCs)_4_ thin films when compared to the bare substrates. Interestingly, this positive effect is intensified with 7 bilayers, since a (GNPs/CNCs)_7_ film depositions on smart paper allows for a 75% OTR decrease and a 59% OTR decrease with PE-coated board, which corresponds to a significant improvement of the oxygen barrier properties. The absolute values of 150 and 600 cm^3^/m^2^·d·bar for smart paper and PE-coated board can be compared with classical samples showing a medium-oxygen barrier: in PET-coated cardboard, a typical value would be 100–150 cm^3^/m^2^·d·bar at 23°C, 50%RH for a coating of 22 g/m^2^ PET (CTP internal data, 2016).

Based on the theory of permeability in the case of two separate laminate layers and using the OTR values measured for the pristine and coated substrates as well as the LbL film thickness measured on the model surfaces (but shown to be in close agreement with the one on the commercial substrates), the permeability of the LbL films was calculated and summarized in Supplementary Table 1 (Crank, 1979). Surprisingly, a dependence of the permeability on the number of bilayers was obtained. It is assumed that the stratified LbL coating only composed of nanoparticles cannot be identified with a homogeneous layer with an inherent, thickness-independent, permeability as is the case for pure polymer layers. The particularly low value of the thickness of the films might also account for this result. However, when considering the 1 ≤ *n* ≤ 7 range, a close to linear improvement of the OTR was obtained.

The permeability of a membrane, such as that of a nanocomposite film is the product of the diffusion coefficient and the solubility. In our case, the coating can be considered as a nanocomposite comprising two types of fillers in a matrix of air. According to the Nielsen model, the presence of the impenetrable GNPs and CNCs results in a longer, more tortuous path for gas molecules to go through, with the result of increasing their diffusion and reducing the overall permeability. This tortuous effect depends not only on the aspect ratio and volume fraction of the fillers but also on the location and orientation of the nanoparticles. It can be expected that the higher the volume fraction, aspect ratio and order parameter, the lower the permeability. In the case of pure CNCs coatings, the permeability data could be fitted using the Bharadwaj model, evidencing the effect of anisotropy on the gas barrier properties (Bharadwaj, 2001; Chowdhury et al., 2019). For the present system, extra complexity arises from the presence of two types of nanoparticles, each one having its own aspect ratio, volume fraction and order parameter, which results in a doubled number of parameters, some of them being unknown. For instance, the total volume fraction was estimated but the individual volume fraction for each type of particle is not known and thus this extra complexity renders almost impossible the fitting of the results to the available models. However, qualitative interpretations of the data can be proposed, given the similarities in the thickness of the coatings deposited onto model or commercial substrates. It can therefore be assumed that a film architecture independent from the substrate was achieved. Accordingly, the very high density (low porosity) of the all-nanoparticle films measured by ellipsometry and neutron reflectivity on model surfaces is most probably conserved in the case of the commercial substrates, which will lead to a high tortuosity and limited oxygen diffusion, even though films of limited thickness (<100 nm) were deposited. Additionally, the measured small thickness increment per bilayer and the SEM observations suggest that both GNPs and CNCs lay flat with their long axis perpendicular to the diffusion direction, which will also maximize the aspect ratio effect on the limitation of oxygen transfer. The observed OTR variations with the number of bilayers can tentatively be attributed to an increase of the tortuous path with the thickness of the film that results in a decrease of the oxygen permeability.

In order to check if the upper layer had any effect on OTR, the deposition of 7.5 bilayers—allowing the upper layer to consist of GNPs—was investigated but no difference with *n* = 7 was observed (Figure 10). This result confirms that the film tortuosity—correlated to particle shapes and film structure—and film thickness are the key parameters governing the oxygen barrier properties at this relative humidity.

## Conclusion

In this study, the ability of thin hybrid multilayered films to be deposited onto various substrates of interest for packaging applications with the goal of enhancing their oxygen barrier properties has been investigated. Structural investigation techniques show that homogeneous films composed of alternating monolayers of CNCs and GNPs nanoplatelets can be built onto the surface of smooth and rigid model substrates. These films, which are of particularly high density, exhibit a thickness that varies linearly with the number of deposited bilayers. Interestingly, these hybrid coatings can also be constructed onto flexible substrates regardless of their hydrophilicity, composition and surface topography. Such versatility arises from the conjunction of the use of the LbL assembly technique and from the intrinsic properties of the two types of nanoparticles used, which were previously shown to strongly interact through electrostatic attractions and hydrogen bonding. OTR measurements demonstrate that these partially bio-sourced all-nanoparticles hybrid thin films improved the oxygen barrier properties when deposited onto flexible paper substrates with up to 75% decrease in the oxygen permeability. This work thus provides an initial perspective for the potential of LbL formed films consisting only of nanoparticles, such as gibbsite nanoplatelets and cellulose nanocrystals, to improve the oxygen barrier properties of flexible paper-based substrates. The adaptability of the process to a wide variety of supports and the very limited thickness needed for substantial barrier properties enhancement implying very small amounts of materials, are strong assets for possible industrial applications.

## Data Availability

All datasets generated for this study are included in the manuscript and/or the Supplementary Files.

## Author Contributions

MC completed the experimental work and analyses. MC, BJ, and LC-A designed the experimental plan and conducted project development. MC and BJ prepared the manuscript and all authors read, revised, and approved the submitted version. BJ, LC-A, DG, and LH conceived the project and contributed to data analysis and manuscript composition.

### Conflict of Interest Statement

The authors declare that the research was conducted in the absence of any commercial or financial relationships that could be construed as a potential conflict of interest.
