# Supplementary material for: Hybrid Gibbsite Nanoplatelet/Cellulose Nanocrystal Multilayered Coatings for Oxygen Barrier Improvement
Source: Front Chem. 2019 Jul 17;7:507. doi: 10.3389/fchem.2019.00507 (PMC6650769; doi:10.3389/fchem.2019.00507)
Supplement: Supplementary file 1 [file Data_Sheet_1.PDF]

Supplementary Material

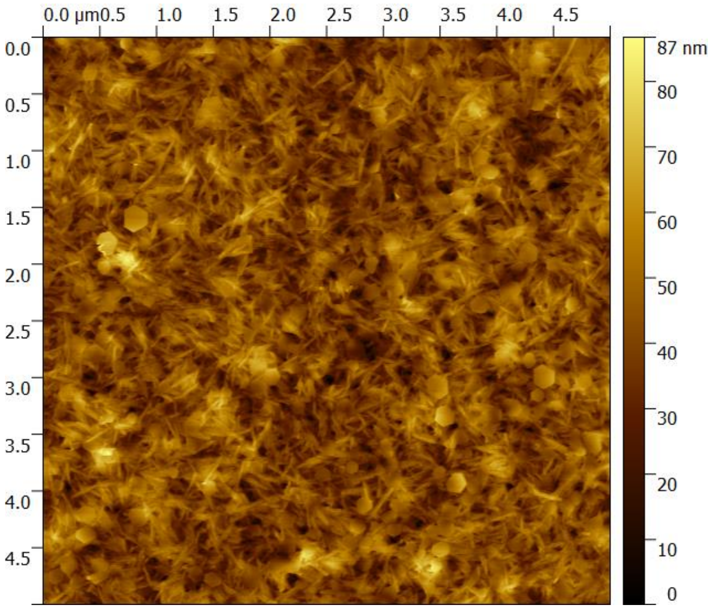

Supplementary Figure 1. 5×5 µm^2 AFM topography image of a $(GNP/CNC)_{4,5}$ multilayered film (GNP-terminated coating) deposited on a Si wafer.

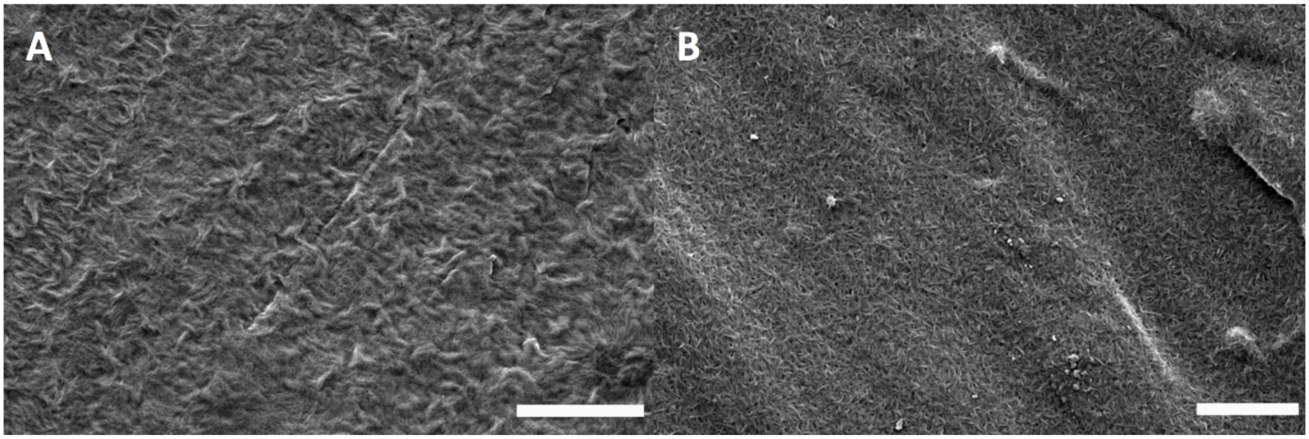

Supplementary Figure 2. SEM images of the PE-LD substrate before (A) and after (B) deposition of a $(GNP/CNC)_4$ multilayered film. Scale bar: 2 µm.

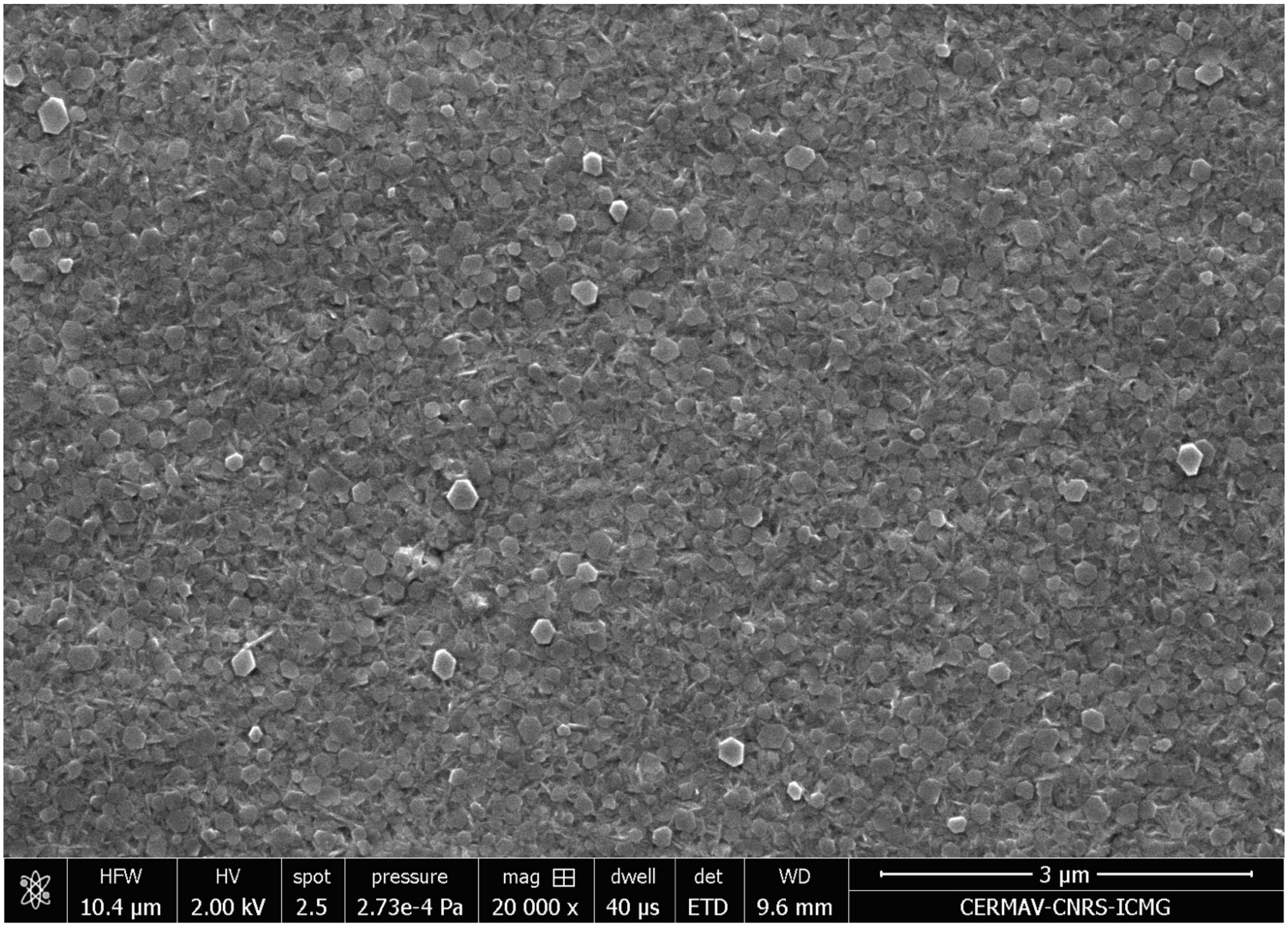

Supplementary Figure 3. SEM images of the PE-LD substrate after deposition of a $(GNP/CNC)_{4,5}$ multilayered film (GNP-terminated coating). The sample was dried at 90°C for 2 minutes at the end of the LbL process.

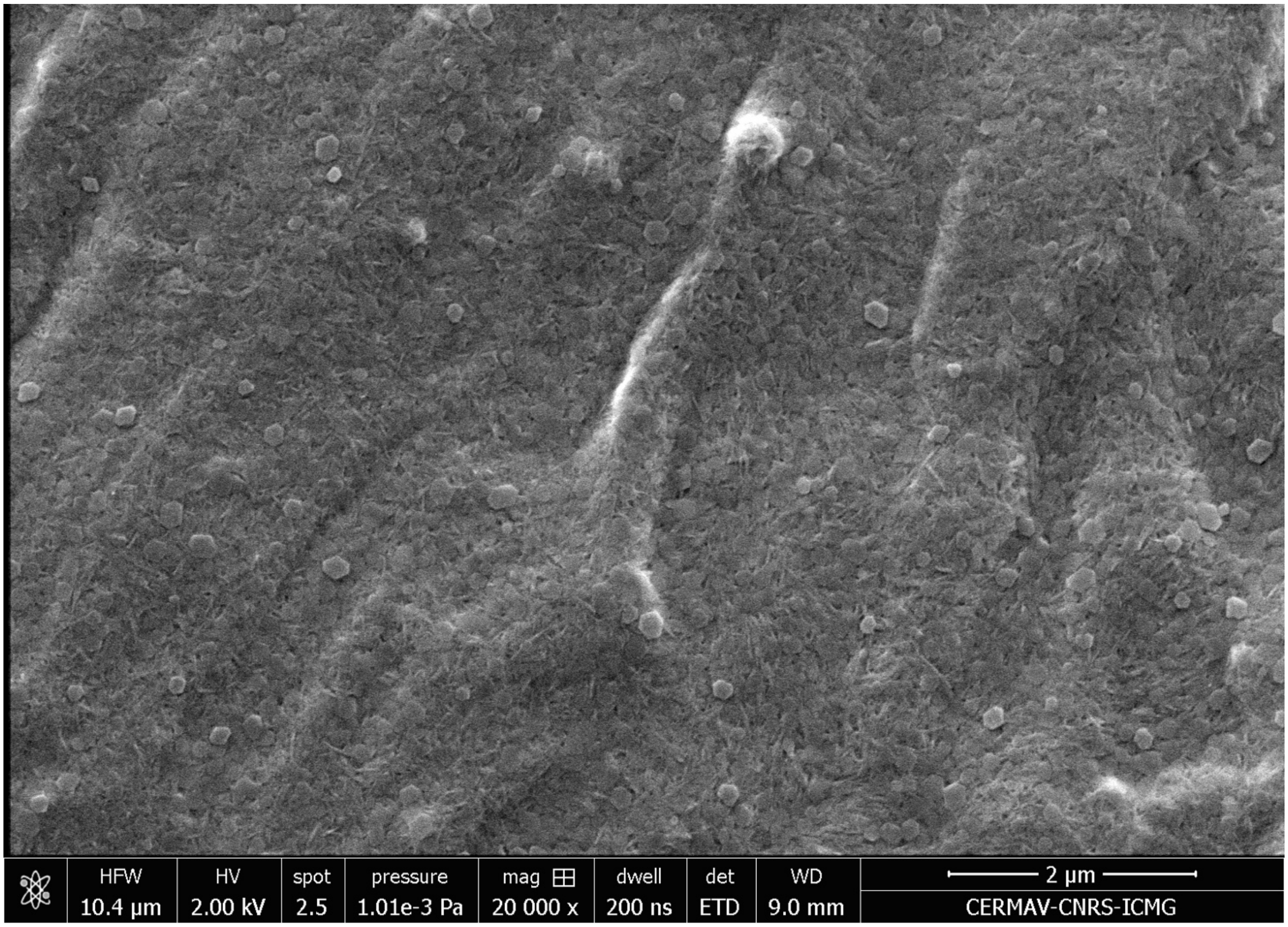

Supplementary Figure 4. SEM images of the Kraft cardboard substrate after deposition of a $(GNP/CNC)_{4,5}$ multilayered film (GNP-terminated coating). The sample was dried at 90°C for 4 minutes at the end of the LbL process.

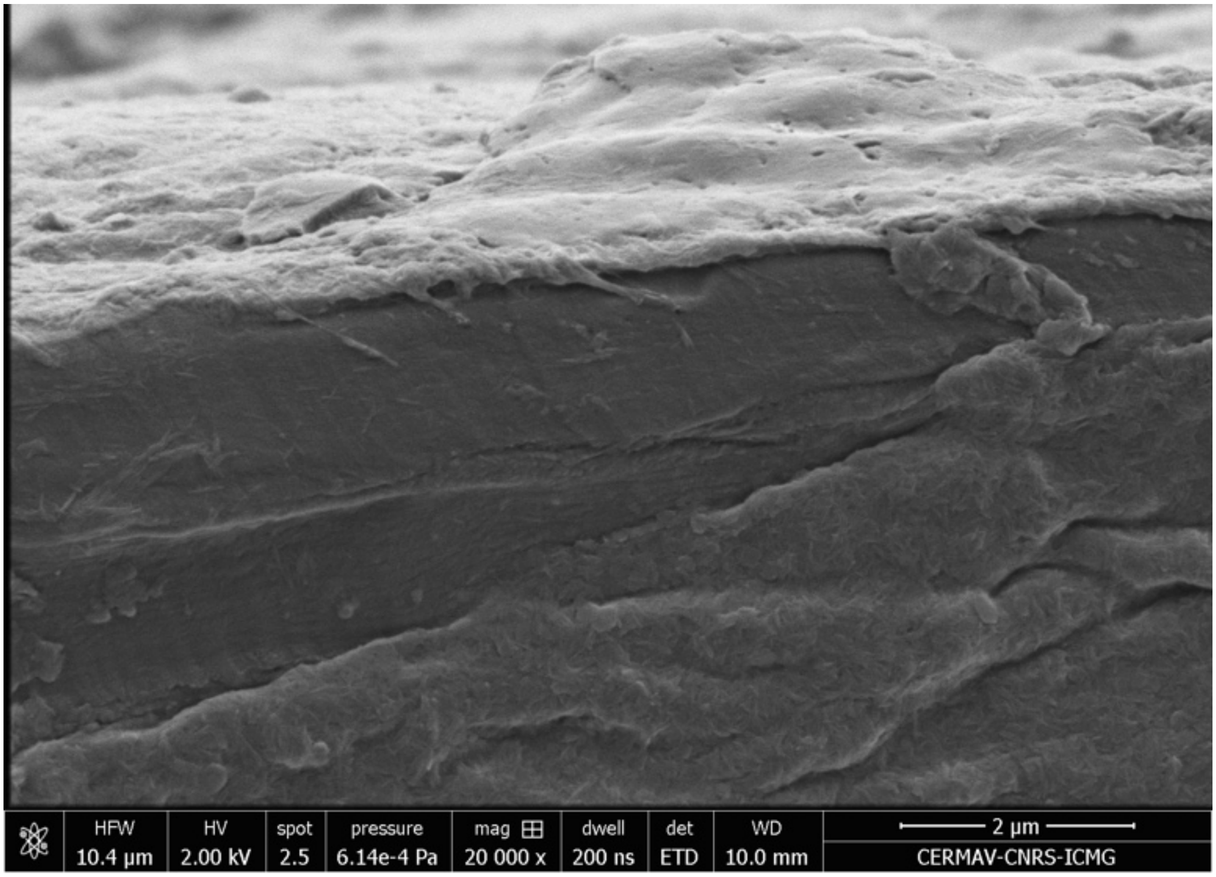

Supplementary Figure 5. Cross-section SEM images of the Kraft cardboard substrate after deposition of a $(GNP/CNC)_4$ multilayered film. The average thickness of the coating layer was estimated to 72±14 nm.

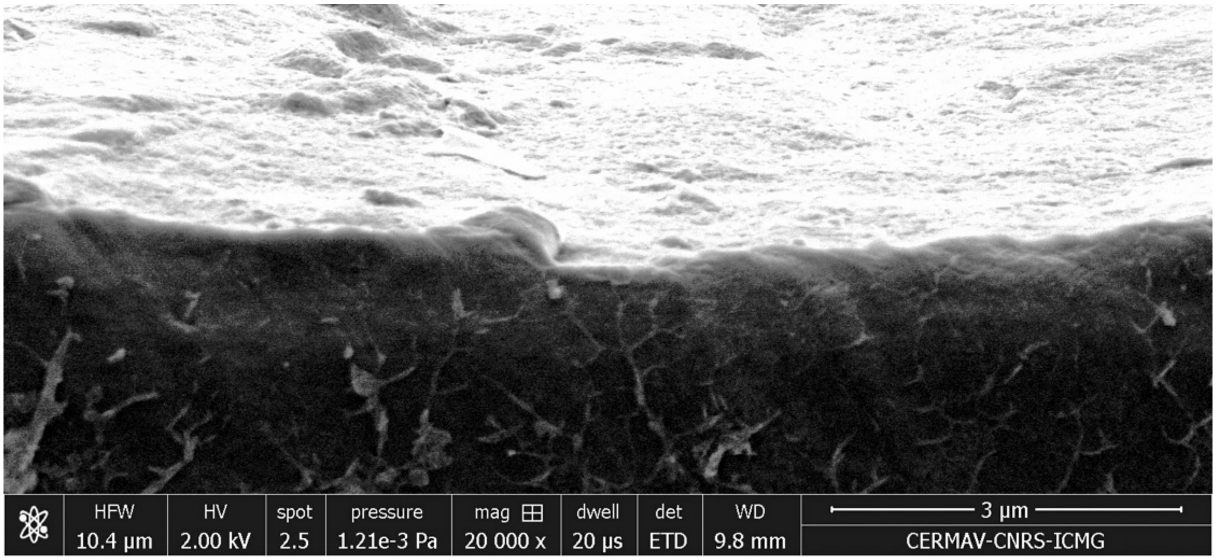

Supplementary Figure 6. Cross-section SEM images of the PE substrate after deposition of a $(GNP/CNC)_4$ multilayered film. The average thickness of the coating layer was estimated to 84±12 nm.

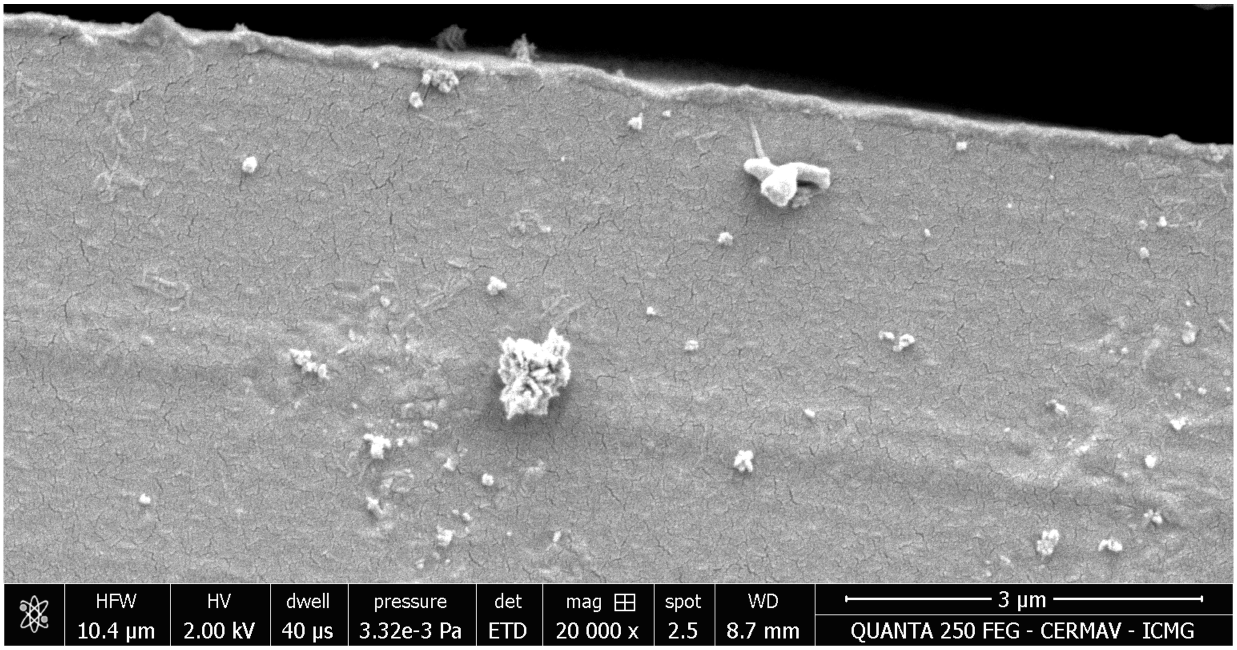

Supplementary Figure 7. Cross-section SEM images of the PE-coated cardboard substrate after deposition of a $(GNP/CNC)_7$ multilayered film. The average thickness of the coating layer was estimated to 122±9 nm.

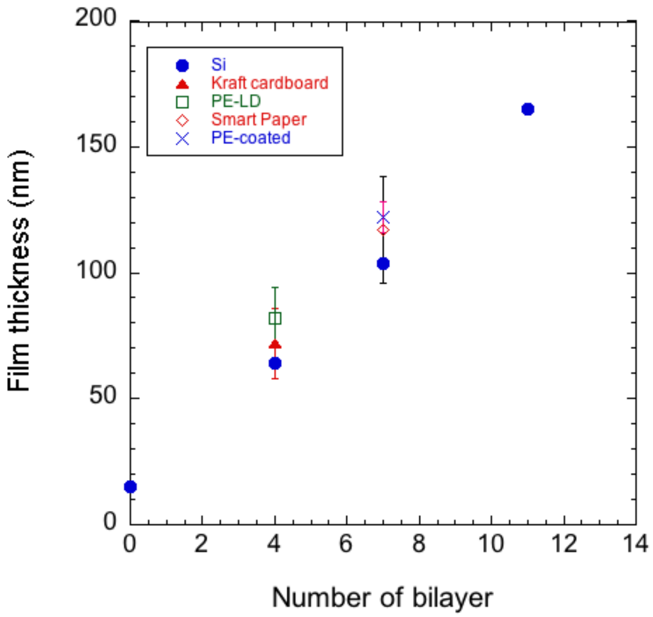

Supplementary Figure 8. $(GNP/CNC)_n$ film thickness as a function of the number of bilayers deposited on a Si wafer (values extracted using spectral reflectance) and Kraft carboard, PE-LD, smart paper and PE-coated cardboard (values extracted from SEM cross-section images).

Oxygen permeability

The oxygen permeability, *P*, of a coated substrate can be expressed as a function of the permeabilities of the LbL film, P_f, and substrate, P_s, through:

$e/P = e_f/P_f + e_s/P_s$ (Eq. 1)

where e, e_f and e_s are the thickness of the coated substrate, LbL film and pristine substrate, respectively (J. Crank The Mathematics of Diffusion, 2d edition, Clarendon Press, Oxford, 1975).

Based equation 1 and using the OTR values measured for the pristine and coated substrates as well as the LbL film thickness measured on the model surfaces (but shown to be in close agreement with the one on the commercial substrates), the permeability of the LbL films were calculated and summarized in Supplementary Table 1.

Number of bilayers	Permeability of the $(GNP/CNC)_n$ film deposited on PE-coated cardboard (cm^3. µm /(m^2.d.bar))	Permeability of the $(GNP/CNC)_n$ film deposited on smart paper (cm^3. µm /(m^2.d.bar))
1	N/A	179
4	192	82
7	127	18
7.5	82	44

Supplementary Table 1. Estimated oxygen permeability of the multilayered films. Pristine substrates have permeabilities of 350 and 225 cm3.µm/(m2.d.bar) for PE-coated cardboard and smart paper, respectively.
